# Digital Health Reimbursement Strategies of 8 European Countries and Israel: Scoping Review and Policy Mapping

**DOI:** 10.2196/49003

**Published:** 2023-09-29

**Authors:** Robin van Kessel, Divya Srivastava, Ilias Kyriopoulos, Giovanni Monti, David Novillo-Ortiz, Ran Milman, Wojciech Wilhelm Zhang-Czabanowski, Greta Nasi, Ariel Dora Stern, George Wharton, Elias Mossialos

**Affiliations:** 1 LSE Health Department of Health Policy London School of Economics and Political Science London United Kingdom; 2 Department of International Health Care and Public Health Research Institute Maastricht University Maastricht Netherlands; 3 Division of Country Health Policies and Systems World Health Organisation Regional Office for Europe Copenhagen Denmark; 4 Digital Health Division Israeli Ministry Of Health Jerusalem Israel; 5 Institute of Public Health Jagiellonian University Krakow Poland; 6 Department of Social and Political Sciences Bocconi University Milan Italy; 7 Harvard Business School Harvard University Boston, MA United States; 8 Harvard-MIT Center for Regulatory Science Harvard University Boston, MA United States; 9 Institute of Global Health Innovation Imperial College London London United Kingdom

**Keywords:** digital health, telehealth, telemedicine, reimbursement, policy, Europe, policy mapping, mapping, pricing, digital health app, application, health care ecosystem, framework, integration

## Abstract

**Background:**

The adoption of digital health care within health systems is determined by various factors, including pricing and reimbursement. The reimbursement landscape for digital health in Europe remains underresearched. Although various emergency reimbursement decisions were made during the COVID-19 pandemic to enable health care delivery through videoconferencing and asynchronous care (eg, digital apps), research so far has primarily focused on the policy innovations that facilitated this outside of Europe.

**Objective:**

This study examines the digital health reimbursement strategies in 8 European countries (Belgium, France, Germany, Italy, the Netherlands, Poland, Sweden, and the United Kingdom) and Israel.

**Methods:**

We mapped available digital health reimbursement strategies using a scoping review and policy mapping framework. We reviewed the literature on the MEDLINE, Embase, Global Health, and Web of Science databases. Supplementary records were identified through Google Scholar and country experts.

**Results:**

Our search strategy yielded a total of 1559 records, of which 40 (2.57%) were ultimately included in this study. As of August 2023, digital health solutions are reimbursable to some extent in all studied countries except Poland, although the mechanism of reimbursement differs significantly across countries. At the time of writing, the pricing of digital health solutions was mostly determined through discussions between national or regional committees and the manufacturers of digital health solutions in the absence of value-based assessment mechanisms. Financing digital health solutions outside traditional reimbursement schemes was possible in all studied countries except Poland and typically occurs via health innovation or digital health–specific funding schemes. European countries have value-based pricing frameworks that range from nonexistent to embryonic.

**Conclusions:**

Studied countries show divergent approaches to the reimbursement of digital health solutions. These differences may complicate the ability of patients to seek cross-country health care in another country, even if a digital health app is available in both countries. Furthermore, the fragmented environment will present challenges for developers of such solutions, as they look to expand their impact across countries and health systems. An increased emphasis on developing a clear conceptualization of digital health, as well as value-based pricing and reimbursement mechanisms, is needed for the sustainable integration of digital health. This study can therein serve as a basis for further, more detailed research as the field of digital health reimbursement evolves.

## Introduction

### Background

The COVID-19 pandemic has significantly accelerated the digital transformation of the health care sector, providing an opportunity to leverage software to prevent, manage, or treat disease [1-6]. Digital health solutions encompass a broad range of technologies that promote, improve, or support health system functioning and the delivery of health care, including electronic health records, telemedicine, mobile health apps, health data analytics, and digital therapeutics [7,8]. These solutions can be used for a range of functions, including web-based consultations with medical professionals, tools and software for remote patient monitoring, real-time updates of algorithms based on patient data, and the delivery of health care interventions. Recent studies have indicated that combining medication with digital health solutions can lead to improved outcomes for a variety of chronic conditions such as type 2 diabetes, cardiovascular diseases, and psychiatric and mental health conditions [9,10].

The adoption of digital health solutions within health systems is subject to various factors, including pricing and reimbursement [8,9,11-13]. Pricing models in health comprise mechanisms such as cost-based pricing (ie, the price of a product is based on the cost of care provided to patients and allowable covered costs), the use of cost-effectiveness thresholds (ie, the price or quality-adjusted life-year is compared with a preset threshold), external price referencing (ie, the price of a product is based on the prices set in other countries), and value-based pricing (ie, the price is based on the value that an intervention adds to the health care process, such as improved health outcomes or reduced costs) [14,15]. The latter approach is recognized as a promising solution to optimize resource allocation and address numerous challenges faced by health systems [11]. National price regulations that incorporate value-based elements, such as the price regulations in Germany, have also been shown to more closely align the prices paid for medical products with their benefit to patients [16]. Value-based pricing also supports evidence-based decision-making in health care procurement by providing a benchmark for what constitutes a high-quality intervention [17]. However, applying a value-based framework to digital health care requires the use of comprehensive frameworks to determine the value of digital interventions and can be challenging because of the continuous improvement of digital health apps through performance data and patient feedback [11,12]. Finally, there is a dearth of data and assessment frameworks in use to evaluate the cost-effectiveness of digital health care [18,19] as well as a scarcity of established pricing models that can be used to streamline the introduction of digital health apps to the health care market [20].

Robust reimbursement mechanisms are vital in facilitating the access and affordability of new health technologies [15], although the reimbursement landscape for digital health in Europe remains underresearched and poorly characterized. Although various emergency reimbursement decisions were made during the COVID-19 pandemic to enable health care delivery through videoconferencing and asynchronous care [21,22], such tools represent only a small set of digital health approaches. Additonally, research so far has primarily focused on the policy innovations that facilitated this outside of Europe. As an example, studies have focused on the Australian government’s recent expansion of Medicare-subsidized telehealth services to facilitate the remote delivery of care and mitigate the risk of virus transmission. Consequently, telehealth services became eligible for reimbursement through the Australian Medicare system and eventually became subject to copayments [22,23]. In the United States, research has highlighted the rapid modification of coverage and payment parity policies by states in response to the COVID-19 pandemic to promote the adoption of telehealth and minimize physical contact, thereby overcoming a significant use barrier [21,24]. In comparison, developments in Europe, particularly in terms of the eligibility of digital health apps (ie, software designed to provide a specific form of therapy with or without the involvement of a health care professional) for reimbursement, remain largely unexplored (a notable exception being Germany, which created the first combined regulation and reimbursement pathway for digital health apps in 2019, which has been described in the literature) [25].

### Objective

This study presents information on the reimbursement practices for digital health in 9 countries within the World Health Organization European Region (WHO/Europe): Belgium, France, Germany, Israel, Italy, the Netherlands, Poland, Sweden, and the United Kingdom. These countries were chosen based on the availability of information on the reimbursement of digital health and feedback received from the Data and Digital Health Unit at WHO/Europe [11,26-29]. We aim to map and compare four distinct reimbursement characteristics across the studied countries: (1) whether digital health solutions are recognized as a reimbursable form of health care, (2) what mechanisms are used to reimburse digital health solutions, (3) how digital health solutions are priced and whether value-based health care frameworks are embedded in that process, and (4) whether any funding is available to reimburse digital health solutions outside of public or private insurance policies.

## Methods

### Policy Mapping Framework

This study uses a policy mapping framework, which has been used and validated by previous research in the fields of autism, disability, and substance abuse policy [30-35]. The policy mapping framework is based on the foundation of a scoping review, which allows for the rapid mapping of the key concepts underpinning a broad research area that is particularly valuable for complex issues that have not been reviewed comprehensively to date [36,37]. The established framework is suited to analyzing the development of health and social policy over time and across multiple layers of governance. In the context of this study, we only sought to collect information on current digital health reimbursement practices; as such, the longitudinal aspect of the policy mapping framework was not applied. We further developed this policy mapping framework from a cross-country analysis lens by presenting both individual country information in tabulated form and cross-country differences narratively. This approach is also supported by previous policy mapping exercises [38-40].

### Data Collection and Analysis

In line with the Joanna Briggs Institute Manual for Evidence Synthesis for Scoping Reviews [41], we searched MEDLINE (Ovid), Embase (Ovid), Global Health (Ovid), and Web of Science on January 20, 2023, and conducted a follow-up search on August 18, 2023, for articles addressing the reimbursement and financing of digital health apps in Belgium, France, Germany, Israel, Italy, the Netherlands, Poland, Sweden, and the United Kingdom. These databases were chosen to cover both the health-specific and interdisciplinary academic fields. To identify gray literature, Google Scholar (first 300 hits [42]) was used. To be eligible for inclusion, a record had to capture a part of the reimbursement or financing pathway of digital health apps in the studied countries. Only records from 2018 onward were eligible for inclusion as this timeframe captures the developments before and during the COVID-19 pandemic in terms of digital health reimbursement and financing as well as the launch of the first country-level reimbursement policy for digital health apps in Germany in late 2019 [25]. The policy mapping framework, in contrast to a traditional scoping review, takes a broader approach to the types of evidence that are eligible for inclusion. Specifically, after searching exclusively for original research and literature reviews, we identified only 14 articles eligible for inclusion, which was too scarce to provide information on existing digital health reimbursement pathways in the studied countries. As such, we expanded the eligibility criteria to include editorials, commentaries, viewpoints, and gray literature as these documents may provide important details of policy developments before these are more rigorously captured in empirical research. We did not directly search for policy repositories as is common practice in the policy mapping framework seeing as we aim to map reimbursement processes rather than the legal basis for reimbursement. Given our specific interest in policy developments in the WHO/Europe region, articles without a focus on the abovementioned countries were excluded.

Table 1 shows the build-up of the search strings for the academic database searches as well as the number of hits per query. The search string was reviewed and validated by an information specialist at the London School of Economics and Political Science Library. The search terms for the supplementary searches in Google Scholar consisted of the phrases “reimbursement of digital health,” “reimbursement of digital therapeutics,” “financing of digital health,” “financing of digital therapeutics,” “digital health tariff,” “digital health pricing,” “telehealth pricing,” “telehealth tariff,” and “financing of telehealth” combined with the respective country. This combination of keywords ensured that less complex forms of digital health solutions (eg, telehealth and telemedicine) and more complex forms (eg, digital health apps or digital therapeutics) were covered in the search string. If the dominant language was a language other than English, we used Google Translate to translate the search phrases into the desired language [43]. To minimize the potential bias introduced through machine translations, experts from the studied countries were asked to assist in searching and interpreting the digital health reimbursement landscape for their respective countries. Additional articles were identified through a review of the references of the studies included through database search. Policy contents and mechanisms were identified from the selected references and reviewed using thematic content analysis. After finalizing the data collection and analysis, country experts reviewed the collected information to validate the findings and, where necessary, add additional expertise and insights. Finally, individual country information was tabulated per studied reimbursement characteristic and the disparities between countries were narratively synthesized.

**Table 1:** Search queries for the respective databases.

Database	Query	Hits, n
MEDLINE (Ovid) Embase (Ovid) Global Health (Ovid)	(digital adj3 (health or medicine or therapeutic? or care)).ti,ab. (ehealth or e-health or mhealth or m-health or telehealth or telemedicine or “health app*”^a^ or telecare or “virtual health” OR “mobile app*”).ti,ab. exp telemedicine/ 1 or 2 or 3 Insurance, Health, Reimbursement/ exp “Costs and Cost Analysis”/ reimburse* OR financ* OR pricing OR price* OR tariff*).ti,ab. 5 or 6 or 7 Belgium/OR France/OR Germany/OR Israel/OR Italy/OR Netherlands/OR Poland/OR Sweden/OR United Kingdom/ (Belgi* OR France OR French OR German* IR Israel* IR Ital* OR Netherlands OR Dutch OR Poland OR Polish OR Sweden OR Swedish OR “United Kingdom” OR Engl* OR Wales OR Welsh OR Scot* OR “Northern Ir*”).ti,ab. 9 or 10 4 and 8 and 11 limit 12 to yr=“2018 -Current”	1004
Web of Science	(TS=(digital NEAR/3 (health or medicine or therapeutic? or care)) OR TS=(ehealth or e-health or mhealth or m-health or telehealth or telemedicine or “health app*” or telecare or “virtual health” OR “mobile app*”)) AND TS=(reimburse* OR financ* OR pricing OR price* OR tariff*) AND TS=(Belgi* OR France OR French OR German* IR Israel* IR Ital* OR Netherlands OR Dutch OR Poland OR Polish OR Sweden OR Swedish OR “United Kingdom” OR Engl* OR Wales OR Welsh OR Scot* OR “Northern Ir*”)	231

^a^Searches were originally performed using straight quotations.

### Ethical Considerations

This study has no inherent ethical implications or considerations.

## Results

### Search Results

**Figure 1:**
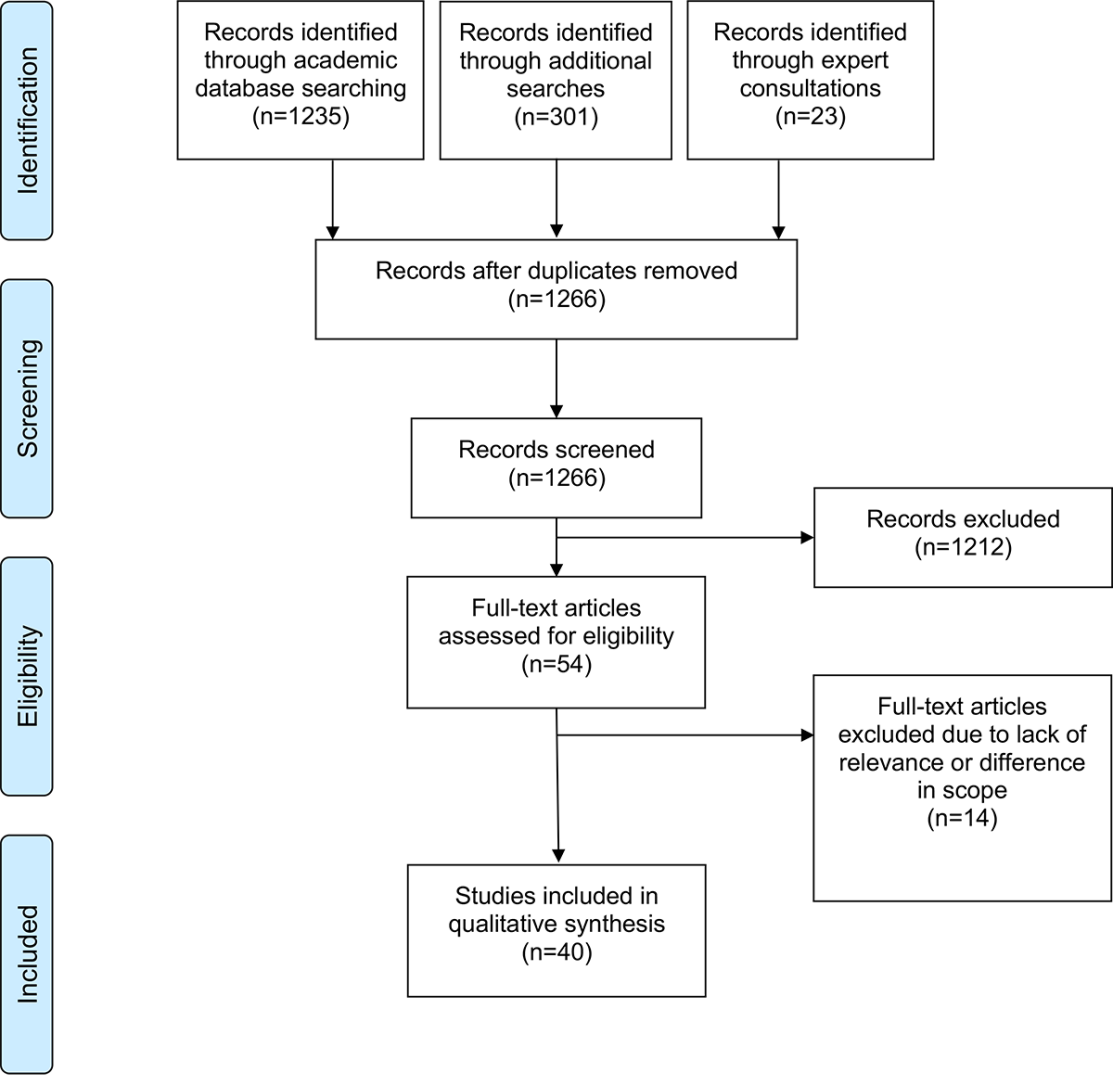
PRISMA (Preferred Reporting Items for Systematic Reviews and Meta-Analyses) flowchart of the screening process.

Our search strategy yielded a total of 1536 records (1235 documents through academic database searching and 301 through supplementary searches). A further 23 records were identified through the country experts. After deduplication, 1266 documents were screened for eligibility, and 2.57% (40/1559) were ultimately included in the analysis. Most documents were excluded because they did not discuss the current reimbursement or financing pathways in place in the studied countries. We included 9 original research articles [11,20,44-50], 5 reviews [8,18,27,51,52], 4 conference abstracts [53-56], 18 gray literature sources [28,57-73], 3 reports [74-76], and 1 commentary [25]. In terms of country focus, 3 documents focused on Belgium [57-59], 8 on France [11,28,51,54-56,60,61], 6 on Germany [18,27,44,52,54,62], 3 on Israel [45,63,76], 4 on Italy [46-48,64], 5 on the Netherlands [8,20,49,65,75], 5 on Poland [50,66-68,74], 3 on Sweden [69-71], and 6 on the United Kingdom [28,53,54,56,72,73]. Figure 1 shows a PRISMA (Preferred Reporting Items for Systematic Reviews and Meta-Analyses) flowchart of the data collection process. The PRISMA-ScR (Preferred Reporting Items for Systematic Reviews and Meta-Analyses extension for Scoping Reviews) checklist is shown in Multimedia Appendix 1. Table 2 shows the details of country-specific digital health reimbursement.

**Table 2:** Country-specific details on the reimbursement and financing of digital health solutions and apps.

Country	Reimbursement characteristics			
	Eligible for reimbursement	Mechanism of reimbursement	Pricing of digital health	Noninsurance reimbursement
Belgium	Yes	Reimbursement of digital health solutions does not focus on reimbursing the solution itself. Rather, the Belgian reimbursement system covers a particular health care trajectory holistically and digital health solutions may be deployed by the practitioner as part of the health care process [57].	Once a digital health solution has reached the highest level of the mHealth^a^ validation pyramid, the pricing is done within the context of the health care process that the app will be integrated into. Each health care process requires its own price determination [58].	The TBM^b^ program aims at contributing to the implementation of new therapies, diagnostic techniques, and preventive methods, which, without government funding, would not make it to the patient due to a lack of industrial interest [59].
France	Yes	Reimbursement of digital health solutions can occur through the centralized pathway of medical devices. Connected medical devices have recently been added to the scope of the French National Authority for Health’s directory of products that qualify for reimbursement within the statutory health insurance [28,54,55]. After the pricing process has been completed, the National Union of Health Insurers adds the digital health solution to a directory of reimbursable products and sets a reimbursement rate for the next 5 y that matches the digital health solution’s clinical benefits assessment [11,54]. Following the success of the ETAPES^c^ experimental program, a reimbursement pathway was introduced in French law under the 2022 Social Security Act (Article 36). This pathway aims to deploy novel remote monitoring apps and requires an evaluation by the French National Authority for Health, much like the existing pathway of medical devices. In case digital health solutions also have therapeutic functions, this pathway can only be applied to the telemonitoring parts of the digital health solution [60]. In 2023, the French Ministry of Health and Prevention introduced an early access pathway for the reimbursement of sufficiently developed digital medical devices (ie, digital health apps) through the PECAN^d^ program [61]. The PECAN program allows 1 y of special coverage by the French health care system, enabling the manufacturer to be reimbursed while they finalize the demonstration of their clinical or organizational benefits.	The Economic Committee for Health Products (CEPS^e^) negotiates the pricing of digital health solution within the statutory insurance with the manufacturer [11,51]. Currently, there are no specific value-based pricing guidelines for digital health solution available [51].	Until December 2022 inclusive, a second reimbursement pathway existed in the form of the experimental program for telemonitoring in France (ETAPES), which focused on the development of telemonitoring approaches in 5 health specialties: heart failure, kidney failure, respiratory failure, diabetes, and implantable cardiac devices [28,56].
Germany	Yes	Digital health apps specifically can be reimbursed under the statutory health insurance as long as they are approved by the Federal Institute for Drugs and Medical Devices and listed in the national digital health directory [18,25,44,52,54]. It is prescribed on a fee-for-service basis [18,27,62].	For the first 12 mo of being listed in the digital health app directory, the manufacturer is generally able to freely set the sales price and pricing model of their digital health app. After 12 mo, the set price is a negotiated price between the manufacturer and the National Association of Statutory Health Insurance Funds [18]. Before the start of the pricing negotiations, the following details have to be clarified [18]: The evidence on general requirements and positive health effects. The results of the studies conducted as part of the possible trial phase. Information on prices for self-payers; Information on prices in other European countries. The complete notification of the Federal Institute for Drugs and Medical Devices about the inclusion of the digital health app in the national directory. The number of activation or prescription codes redeemed for the digital health app in the period from inclusion in the national directory to 5 d before submission.	Financial support for the development of digital health apps can be obtained from the German Innovation Fund, funded by the German Health Insurance (Gestzlicher Krankenversicherung) [27].
Israel	Yes	Health maintenance organizations are obligated to provide the services described in the National List of Health Services to their insured population [63,76]. The list is updated annually by an appointed Public Committee, with additional budget allocated to these new additions. As the national list is part of the health insurance law, the technologies not considered as stand-alone technologies but as entitlement to the medical service provided by these technologies. The health maintenance organizations may choose to use digital health solutions to provide an existing entitlement, instead or in parallel to the more traditional methods. Health maintenance organizations can also decide to purchase digital health solutions as part of a service that is not included in the national list. In this case, they fund it through their internal basket or through the complementary insurances [63].	When a new technology is added to the national list, its price is estimated by subcommittee adjacent to the public committee and used for budgeting purposes [45,76]. However, the actual price of the technology is negotiated between each of the health maintenance organizations and the manufacturer [63].	Other funds that are available to reimburse digital health solutions outside insurance policies mostly include Ministry of Health grant programs specifically aimed at supporting the development and implementation of digital health solutions. These funds are used to support the development of new digital health solutions or to help fund the adoption and implementation of existing solutions [63].
Italy	Yes	Digital health solutions can be reimbursed under the national health system [46], although the reimbursement procedures of digital health apps represent an open challenge and is open to multiple approaches [47]. All regions in Italy adopted tariffs for telehealth and matching reimbursement procedures for all modes of service delivery (digitally supported or in person) [64]. These tariffs should follow a payment parity mechanism, indicating health care providers are paid a fixed amount/patient, regardless of the services provided and their mode of provision [64].	Digital health solutions that enhance the current health care and therapies process may be merged and embedded into an updated price list of existing services. Pricing protocols for digital health solutions that require a fee-for-subscription attached to a drug or medical device have not yet been established [47].	Pilot projects within the national health system promote the integration of digital health solutions into the delivery of health care through public health services [48].
Netherlands	Yes	The Dutch Healthcare Authority has published guidance documents to help health professionals in the Netherlands distinguish between clinical medical apps and assistive health apps. This distinction is important in the Dutch health care context to determine whether a digital health solution has to be reimbursed under individual health insurance companies (in the case of use in primary care, home, or community settings) or whether they can be reimbursed by the basic health insurance package under the diagnosis-related groups (in the case of hospital-based specialist care) [65].	Pricing of (digital) health solutions in the Netherlands currently involves a negotiation between the health insurers, health providers, the Dutch Healthcare Authority (NZa^f^), and the Dutch Health Institute (Zorginstituut Nederland), though no specific guidelines for the pricing of digital health solutions have been established [49,75].	The Dutch Healthcare Authority offers a financing mechanism for “promising types of healthcare” until 2023 inclusive, which cover treatment options currently not yet covered by the basic health insurance [65]. In addition, some digital health solutions are made available through sponsorships from nongovernmental organizations (eg, Alzheimer Netherlands sponsoring 2 digital health apps for dementia) [8,20].
Poland	No	A reimbursement pathway within the National Health Fund for digitally delivered health services was created as part of an emergency COVID-19 pandemic policy. The scope of digital health in Poland is currently limited to tool of digital consultation between health care professionals and patients, consultations between health care professionals, as well as the e-prescription system [50]. This situation is further strengthened by the limitation that reimbursable digital health care should be performed by a health care professional whose services are already covered under the National Health Fund [66].	Health care services tariffing involves the President of the National Health Fund, the Tariffing Council, and the Minister of Health [74]. The Tariffing Council is responsible for providing an opinion on the determination of the tariff of the service, whereas the Minister of Health is responsible for approving the tariffing plan. The report on the determination of the tariff of the service includes a description of the health care service subject to tariffing, an analysis of demand and current and desired supply of the health care service subject to tariffing, a description of the manner and level of financing of the health care service subject to tariffing in other countries, an analysis of cost data, a draft tariff of the service, an analysis of the financial effects on the health care system, and other available data necessary to determine the tariff of the service. The Tariffing Council is required to issue an opinion on the determination of the tariff of the service within 30 d of receipt of the report. The Supreme Audit Office notes that the pricing of individual telemedicine services reimbursed by the National Health Fund usually takes much less time than the pricing of other services [67].	Although no concrete alternative financing pathways are laid out, the Program for the Development of eHealth in Poland for the years 2022-2027 indicates that funds can be obtained for this purpose from programs, such as the Regional Operational Programme, Operational Programme Infrastructure and Environment, National Recovery Plan, and the Digital Europe Programme [68].
Sweden	Yes	For the purpose of streamlining the remuneration of digital health care, Sweden drafted recommendations in 2019 on what regions or local authorities should be reimbursed for if a citizen from another region seeks digital health care within their region [69].	Each region is responsible for the price-setting of digital health care services and the corresponding copayments for patients. However, patients are not limited to only seek health care in their region of residence, resulting in a complicated system with different prices for digital health care outside the county and for physical care within the region [70].	Public funding for early-stage innovations can be acquired through Vinnova, Sweden’s innovation agency [71].
United Kingdom	Yes	Once a digital health app receives a positive recommendation from the NICE^g^, the app becomes eligible for purchase by the integrated care boards pending negotiations [28,56]. Delivery of digital health apps to patients is free of charge at the point of service [72].	In their assessment, NICE provides recommendations on a value-based pricing for a digital health app. However, pricing negotiations occur individually with the 42 integrated care boards (the replacement of the clinical commissioning groups) [28,53,54,56].	In England, the MedTech funding mandate can reimburse the costs of using digital health apps for a duration of up to 4 y, though a positive assessment of the NICE is required to be eligible for this funding scheme [73]. The NHS^h^ Innovation Accelerator aims to fast-track digital innovations into the NHS by supporting high-impact, evidence-based interventions and providing bursaries for scaling across the NHS. The Innovation and Technology Tariff has been introduced in 2017 to foster the adoption and centralize funding of 6 innovations deemed suitable for NHS-scale deployment (one of which is a digital health intervention) [53].

^a^mHealth: mobile health.

^b^TBM: Toegepast Biomedisch onderzoek met een primair Maatschappelijke finaliteit.

^c^ETAPES: Expérimentations de Télémédecine pour l’Amélioration des Parcours en Santé.

^d^PECAN: Prise en Charge Anticipée Numerique des Dispositifs Médicaux.

^e^CEPS: Comité Economique des Produits de Santé.

^f^NZa: Nederlandse Zorgautoriteit.

^g^NICE: National Institute for Health and Care Excellence.

^h^NHS: National Health Service.

### Eligibility and Mechanism of Reimbursement Through Insurance

All studied countries except Poland allowed for some reimbursement of digital health solutions. However, the reimbursement mechanisms differed substantially across countries, which can be attributed in part to the differences in how the health systems are financed. Two reimbursement archetypes could be derived from the studied countries: countries either reimbursed the digital health solution itself (France, Germany, Italy, the Netherlands, Poland, Sweden, and the United Kingdom) or reimbursed the clinical pathway that the digital health solution is part of (Belgium and Israel). Further variation was observed across the studied countries in terms of how the digital health apps were classified. Belgium, France, and Israel classify digital health solutions under the traditional paradigm of medical devices, whereas Germany, Italy, the Netherlands, Poland, Sweden, and the United Kingdom recognize digital health solutions as their own classification. Italy was the only country to implement an explicit system of parity for the reimbursement of digital health solutions. This means that health providers are paid a fixed fee regardless of whether health care is delivered face-to-face or through digital health.

### Pricing of Digital Health Solutions or Apps

Pricing mechanisms were found to be at different stages of development across countries. France, Germany, Israel, and Poland each have a dedicated committee that decides on the price of digital health solutions or apps within their respective national insurance systems, although only Germany reports a concrete framework upon which the prices of digital health apps are based. In contrast, Sweden and the United Kingdom delegate pricing negotiations to the regional level, with each region being responsible for reaching an agreement with the digital health manufacturer. The Netherlands exhibited a combination of both approaches, depending on whether a specific digital health solution was included in their mandatory health insurance package or the optional insurer-determined insurance package. In contrast, Belgium and Italy determine the price of digital health solutions in the context of the health care process in which they are deployed. Italy reported having no concrete pricing framework in place for digital health apps even though they are deployed on a fee-for-service basis. Despite using differentiated approaches, all countries adopted a variation of cost-based pricing for digital health except for Belgium, where it is integrated in a value-based pricing system and Germany, where price negotiations need to be based on the demonstrated value of the digital health solution.

### Financing Digital Health Solutions or Apps Outside Insurance

The studied countries reported an array of options for financing digital health solutions that were not reliant on their inclusion in health insurance packages. France offered an experimental program for digital health solutions in the fields of heart failure, kidney failure, respiratory failure, diabetes, and implantable cardiac devices until January 2023. Belgium, Germany, Israel, Italy, the Netherlands, Sweden, and the United Kingdom offer innovation grants that finance treatment options currently under development and are not yet covered by their respective national health insurance frameworks, which can cover digital health solutions. However, in Germany, digital health apps that are approved via Germany’s fast-track process, which combines regulation and reimbursement, have to be directly reimbursed in the statutory health insurance system. Furthermore, certain nongovernmental organizations in the Netherlands offer access to disease-specific digital health solutions outside of insurance packages. In this scenario, they are purchased by the nongovernmental organization and distributed to its members free of charge. Poland was the only country that did not report any concrete funding mechanism for digital health solutions.

## Discussion

### Principal Findings

Although there is nascent literature on national reimbursement practices vis-à-vis digital health solutions, this study is, to the best of the authors’ knowledge, the first to compare reimbursement pathways across countries in the WHO/Europe region with and without specified digital health pathways and to do so for a broader set of digital health tools and approaches. Our findings reveal that the reimbursement pathways for digital health are varied and that value-based pricing frameworks are rare. Although this can be partly attributed to the distinct systems for financing health care in the examined countries, it also emphasizes how the absence of a consistent definition and classification of digital health can contribute to disparate policy and implementation approaches. The present conceptualization of digital health solutions can encompass a range of meanings, including technology, user experience, individual service, product, or process, and can be viewed as part of the broader ecosystem of health services [2]. Although this expansive definition allows for significant flexibility in integrating digital health into existing health care delivery and reimbursement frameworks, it can also lead to uncertainty regarding where and how digital health is meant to fit within a health system. For instance, digital health solutions are regarded as stand-alone health care products in France, Germany, Italy, the Netherlands, Poland, Sweden, and the United Kingdom, whereas they are framed as tools to deliver traditional health care in Belgium and Israel. Furthermore, Belgium, France, Germany, Italy, and the United Kingdom have specific policy frameworks in place that outline the concept of digital health apps, whereas the other countries categorize digital health apps under either the broader digitalization of health care or medical devices [18,28,56,60,65].

Overall, the observed differences may be explained by the novelty and unprecedented nature of digital health tools and approaches, especially in light of how change resistant the health care sector can be [77]. Another contributing factor may be the lack of digital health literacy among both clinicians and patient users and the necessity (but not sufficient) for adoption training of the health workforce and policy makers to understand the scope, potential benefits, and limitations of these digital transformations [5,78,79]. Furthermore, the acute need to act during the COVID-19 pandemic resulted in divergent policies being implemented across Europe [38,40]. When combined, these factors may help explain why disparate digital health reimbursement policies are currently in place across the studied countries.

In the specific context of digital health apps, we found 2 broad categories of approaches toward reimbursement in the studied countries. On the one hand, digital health apps may be reimbursed per use cycle (ie, the period in which the digital health app must be used to produce positive health benefits). This fee-for-service approach has historically been easy to develop and implement, reflects the actual number of services rendered, and can create an incentive for health care providers to increase access to and use of health care services [80]. However, this reimbursement approach has a significant and oft-cited disadvantage, namely providing health care professionals with the incentive to induce demand [81,82]. In the context of digital health care, which can function autonomously and asynchronously, this risk may be exacerbated as health professionals no longer have to consider their own time constraints in prescribing this form of health care (although it could be tempered when a digital health solution is expected to be a substitute for billable services by a clinician). Consequently, a digital health economic paradox can arise, namely that digital health care has the potential to reduce health care costs relative to in-person services, but can equally inflate health care spending if poorly implemented, although this is dependent on the reimbursement methods applied and whether digital health tools serve as a complement or substitute for in-person care—or a combination of the 2. On the other hand, reimbursement for digital health apps may be included as part of the remuneration of a larger health care process, which is more compatible with diagnosis-related groups or global budget approaches to paying for health care. Both these approaches may be more suited than fee-for-service for realizing the potential for digital health care to reduce costs, as they characteristically introduce incentives to improve efficiency in processes and in the allocation of resources.

Furthermore, reimbursement parity for digital health solutions was explicitly introduced in Italy, although the Belgian and Israeli reimbursement approaches could produce a degree of parity as well. Parity systems are considered enablers for the uptake of digital health [21,24], although concerns exist that parity systems could impede the development of innovative care delivery models, which could limit the potential of telehealth to address high health care costs and complicate the introduction of value-based models [24].

Among the countries examined, the pricing of digital health solutions and apps was mostly determined through discussions between committees at the national or regional level and the digital health app developers and manufacturers. However, it is presently unclear what criteria must be met to participate in these committees and whether the designated institutions have the necessary capabilities to assess the worth of digital health solutions or apps [11,83]. Only France, Germany and the United Kingdom have reported the use of value-based frameworks with clear information requirements to help stakeholders navigate the price negotiations for digital health apps. These 3 countries are at a more advanced stage of digital health implementation and both patients and professionals have more experience with digital health apps and their effects, which is an important foundation for the introduction of value-based reimbursement models [17].

We found that, except for Poland, there were many opportunities for financing digital health solutions and apps outside of insurance-based frameworks in the studied countries. However, there were discrepancies in the specificity of these funding opportunities, as it was unclear whether they targeted digital health specifically or health innovations more generally. Nonetheless, such funding initiatives can alleviate the financial risk for health insurers and allow patients, professionals, and insurers to become accustomed to the use and effects of digital health solutions and apps, which is a key factor in their uptake [84].

### Limitations

Some limitations of this study need to be considered. As is common with scoping reviews, the quality of the included studies was not assessed, which should be considered when interpreting the results. However, as the aim of this study was not to validate methodological rigor to ascertain confidence in the data synthesis but rather to collect information about reimbursement processes in different countries, the absence of a quality assessment does not inhibit the validity of this study. In fact, the information collected did not solely rely on scientific articles as country experts also ensured that the collected information was complete and correct. We also recognized the possibility of selection bias and failed to capture all relevant studies as only 3 academic databases and Google Scholar were used, and the search strategy was not exhaustive. This study focused on 9 countries in the WHO/Europe region, which may not reflect the realities of other regions or countries. Furthermore, the lack of a uniform definition and classification of digital health across countries may have influenced the interpretation and comparison of the reimbursement pathways. Therefore, caution should be exercised when interpreting and generalizing the findings of this study. Moreover, the rapidly evolving nature of digital health and its reimbursement pathways means that the information presented in this study may become outdated relatively quickly. Finally, this study did not explore the potential impact of reimbursement policies on patient outcomes, which could be an area of future research.

We have identified several avenues for future research. First, the composition of committees that determine the price of the digital health solutions in the studied countries could be better characterized and assessed. Second, the expertise requirements to adequately assess the value and price of digital health solutions could be further investigated and potentially pooled into a comprehensive framework. Third, a comprehensive, value-based, and value-sensitive framework for the assessment of digital health apps should be developed. Although some research in this area has already been conducted [19,85-87], no comprehensive assessment of how digital health apps generate value across different levels of health systems has been conducted so far. Fourth, a comparative assessment of health expenditure (eg, in the management of a particular disease) after the introduction of digital health care in the studied countries can offer a more sophisticated insight into how digital health interacts with different reimbursement systems to affect overall health expenditure. Fifth, it is unclear whether these findings apply to the broader spectrum of novel health technologies. Digital health is a particular type of innovation in health care and future work can explore how these findings translate to, for instance, the growing role of artificial intelligence in health care, where artificial intelligence–based digital health solutions represent a unique subset of digital health products. Sixth, future research should investigate how digital health solutions or apps would be reimbursed in case of cross-country health service delivery and how these reimbursement practices interact with current international and European Union legislation. Finally, future research should investigate how digital health policy has evolved over time across multiple layers of governance (eg, international, national, and regional where applicable) and even before the COVID-19 pandemic to better understand the extent to which the pandemic accelerated the development of digital health policy holistically and how that affected the formation of reimbursement-specific policy for digital health.

### Conclusions

The studied countries have been pursuing heterogeneous approaches to the reimbursement of digital health care. Although no approach is inherently superior, a fee-for-service approach might encourage more prescribing of certain digital health solutions or apps, which may be desirable to increase digital health use for purposes such as screening, yet it equally opens the possibility of supplier-induced demand and thus inflating health care expenditure. Ultimately, reimbursement policies should aim to stimulate the value-based integration of digital health into the health care ecosystem to promote equitable access to innovative health care technologies and improve health outcomes for patients. A clearer understanding of reimbursement and its accompanying incentives at present will help to shape more thoughtful, value-based reimbursement policies going forward. This study can therein serve as a basis for further, more detailed research as the field of digital health reimbursement evolves.

## Appendix

Multimedia Appendix 1 PRISMA-ScR (Preferred Reporting Items for Systematic Reviews and Meta-Analyses extension for Scoping Reviews) checklist.

## Abbreviations

PRISMA: Preferred Reporting Items for Systematic Reviews and Meta-Analyses
PRISMA-ScR: Preferred Reporting Items for Systematic Reviews and Meta-Analyses extension for Scoping Reviews
WHO/Europe: World Health Organization European Region

## Data Availability

No primary data were collected for this study.
